# High mutation load, immune-activated microenvironment, favorable outcome, and better immunotherapeutic efficacy in melanoma patients harboring *MUC16*/CA125 mutations

**DOI:** 10.18632/aging.103296

**Published:** 2020-06-03

**Authors:** Qinghua Wang, Yichen Yang, Meng Yang, Xiangchun Li, Kexin Chen

**Affiliations:** 1 Department of Epidemiology and Biostatistics, National Clinical Research Center for Cancer, Key Laboratory of Molecular Cancer Epidemiology of Tianjin, Tianjin Medical University Cancer Institute and Hospital, Tianjin 300060, China; 2 Tianjin Cancer Institute, National Clinical Research Center for Cancer, Key Laboratory of Cancer Prevention and Therapy of Tianjin, Tianjin Medical University Cancer Institute and Hospital, Tianjin 300060, China

**Keywords:** *MUC16*, tumor mutation load, immune microenvironment, survival outcome, immunotherapeutic efficacy

## Abstract

Immunotherapies have dramatically improved survival outcome for patients with melanoma. *MUC16* encodes cancer antigen 125 (CA125), which is frequently mutated in melanoma. In this study, we correlated the *MUC16* mutational status with the following: tumor mutation burden (TML), multiple immune-related signals in microenvironment, deregulated pathways, survival outcome, and immunotherapeutic efficacy. We found that patients with *MUC16* mutations had significantly higher TML than those without it. Enriched pro-inflammatory CD8 T cells and M1 macrophages, enhanced interferon gamma (IFNγ) and T cell-inflamed signatures, and increased cytolytic activity were associated with *MUC16* mutations. Immune-suppressive M2 macrophages were enriched in patients with wild-type *MUC16*. Immune checkpoints expression (e.g., *PD-L1*, *PD-1* and *CTLA-4*) was also elevated in patients with *MUC16* mutations. Immune response relevant circuits were among the top enriched pathways in samples with *MUC16* mutations. Patients with *MUC16* mutations exhibited a significantly better prognosis. For patients who received immunotherapy, the presence of *MUC16* mutations was associated with a better response rate and survival outcome in male patients but not in female or overall patients. These findings provide new implications for tailoring immunotherapeutic strategies for melanoma patients.

## INTRODUCTION

Melanoma is characterized by rapid progression and poor survival [1]. Early-stage localized melanoma patients could be effectively treated through surgical resection, but survival outcome for patients with distant metastases is always less favorable [2]. Recently, kinase inhibitors (e.g., vemurafenib, dabrafenib and trametinib) that target specific pathways have been approved by the Food and Drug Administration (FDA) [3]. Although the noteworthy improvement of antitumor response to these agents has been observed, they are rarely durable [2].

Owing to the emergence of immunotherapy, especially immune checkpoint inhibitor (ICI) therapy, prognosis for melanoma patients has dramatically improved [4–6]. However, only a subset of patients could demonstrate a remarkable response to ICI therapy. The pivotal point of this problem is the lack of effective indicators to identify patients who are more responsive to immunotherapy agents. The current broadly used biomarker of immune treatment response is tumor mutation load (TML). Other multiple microenvironment-based factors, such as immune checkpoints expression, proportion of tumor infiltration lymphocytes (TIL), and interferon gamma (IFNγ) signature, also play vital roles in response to immunotherapy. Patients who harbor fewer markers may benefit less from such treatment. This raises the question whether there exist other factors that simultaneously affect more than one of the above listed biomarkers, which could provide better predictive value for immunotherapy.

*MUC16*, which is a member of the mucin family and encodes cancer antigen 125 (CA125). *MUC16* was determined as the monitor indicator for diagnosis of gynecological cancer [7–9]. Several recent studies have reported that *MUC16* could inhibit antitumor immune responses by attenuating natural killer (NK) cells and promoting regulatory T cells activity [10–12]. Meanwhile, other studies demonstrated that *MUC16* may be implicated in enhancing the activity of pro-inflammatory pathways in tumor [13, 14]. Our previous study reported the association of *MUC16* mutations with high TML and favorable outcome in gastric cancer (GC). Furthermore, our results revealed that mutated *MUC16* has important implications for immunotherapy [15]. However, owing to the limited number of GC samples from patients who received immune treatment, we could not validate the relevance of these results. To our knowledge, the effect of *MUC16* mutations on melanoma TML, microenvironment, prognosis, and immunotherapeutic efficacy has not yet been investigated.

In this study, we explored whether the presence of *MUC16* mutations was associated with TML, tumor-immune microenvironment, survival outcome, and ICI treatment efficacy in melanoma. Evidence derived from our study would have implications for guiding immunotherapy.

## RESULTS

### *MUC16* mutational status of melanoma

**Figure 1:**
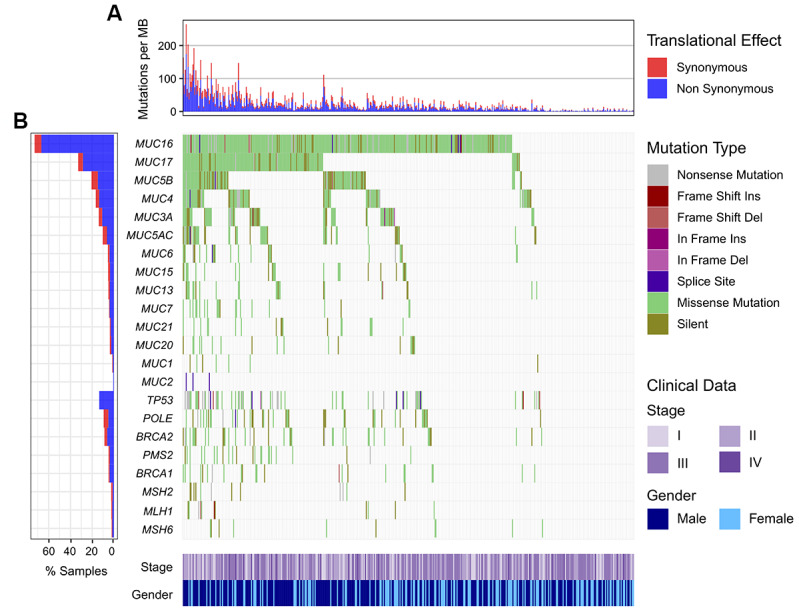
**Mutational patterns of *MUC16* and mucin family members in relation to DNA repair-related genes in the TCGA cohort.** (**A**) Numbers of mutations per megabase in each sample. (**B**) Representation for mutation patterns of mucin and DNA repair genes.

*MUC16* was one of the most frequently mutated genes in melanoma. Of the 467 samples in the Cancer Genome Atlas (TCGA) cohort, 341 (73.1%) harbored *MUC16* mutations. Plenty of frequently mutated genes were correlated with TML in TCGA, and presence of *MUC16* mutations showed the most significant correlation (*P* = 2.25E-44; Supplementary Figure 1). We found that patients with mutated *MUC16* had a significantly higher TML than those without it (Figure 1A). Mutation distribution of *MUC16* and its family members in relation to genomic integrity maintenance genes was illustrated in Figure 1B. Waterfall plot showed that patients who harbored *MUC16* mutations also had some mutations in genome repair genes (145 of 341, 42.5%; Figure 1B). Consistent results and mutational patterns of mucin family members and genome repair genes in the International Cancer Genome Consortium (ICGC) cohort were exhibited in Supplementary Figure 2.

The difference in TML between the 2 cohorts was not statistically significant (median TML: TCGA cohort 3.78 vs. ICGC cohort 4.27; Wilcoxon rank sum test, *P* = 0.11; Supplementary Figure 3).

### *MUC16* mutations are associated with high TML in both cohorts

**Figure 2:**
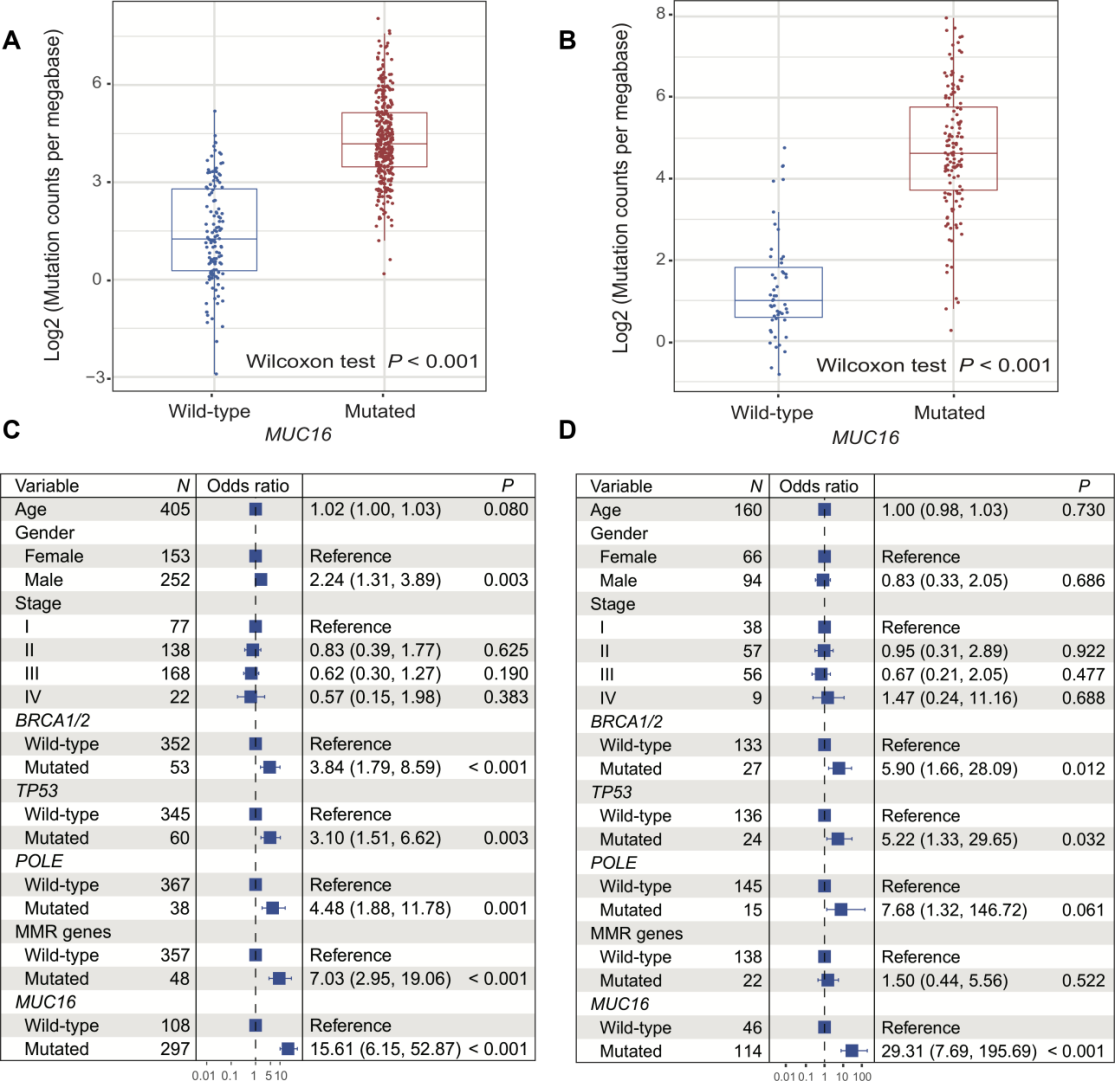
**Correlation of *MUC16* mutations with tumor mutational load in 2 cohorts.** (**A**, **B**) Mutation burden of melanoma samples with and without *MUC16* mutation (left: TCGA; right: ICGC). (**C**, **D**) Multivariate logistic regression models were conducted to explore association of *MUC16* mutations with TML (left: TCGA; right: ICGC).

In the TCGA cohort, melanoma patients with *MUC16* mutations had a significantly higher TML than those without *MUC16* mutations (median TML: 4.19 vs. 1.25; Wilcoxon rank sum test, *P* < 0.001; Figure 2A). In *MUC16* mutated patients, we found that *BRCA1/2* (56 [16.4%] patients with mutations), *TP53* (57 [16.7%] patients with mutations), *POLE* (44 [12.9%] patients with mutations), and MMR genes (total 49 [14.4%] patients with mutations) were significantly co-mutated (Fisher exact test, all *P* < 0.01; Supplementary Table 1). Mutations in these genes caused a significantly higher mutation load (OR > 3, *P* < 0.01; Figure 2C). TML could be suitably divided into high and low subgroups with a cutoff value of 4.22 (Supplementary Figure 4). To rule out the possibility that higher TML was generated by mutations in genome repair genes rather than directly by *MUC16* mutations, we performed multivariate logistic regression model with mutations in DDR and MMR genes, and clinical confounding factors taken into consideration. Association of *MUC16* mutations with higher TML was still statistically significant after adjusting for these confounding variables (OR: 15.61, 95% CI: 6.15-52.87, *P* < 0.001; Figure 2C).

Additionally, high TML was also observed in tumor samples with *MUC16* mutations of other immune activated tumor types (e.g., cancers of the lung, colorectal, kidney, bladder, and head and neck) in the TCGA cohort (Wilcoxon rank sum test, all *P* < 0.001; Supplementary Figure 5).

Of the 183 melanoma patients in the ICGC cohort, the significantly high TML was also observed in patients with *MUC16* mutations (median TML: 4.63 vs. 1.01; Wilcoxon rank sum test, *P* < 0.001; Figure 2B). Genomic integrity maintenance genes, including *BRCA1/2* (26 [19.7%] samples with mutations), *TP53* (27 [20.5%] samples with mutations), *POLE* (16 [12.1%] samples with mutations), and MMR genes (total 22 [16.7%] samples with mutations) were also significantly co-mutated in these *MUC16* mutated patients (Fisher exact test, all *P* < 0.01; Supplementary Table 1). Multivariate logistic regression model that included these gene mutations and clinical variables was performed to control confounders. In this model, the association between *MUC16* mutation and high TML was still statistically significant (OR: 29.31, 95% CI: 7.69-195.69, *P* < 0.001; Figure 2D).

In addition to association of *MUC16* mutation status with TML, we also discovered that the total count of *MUC16* mutations was associated with high TML in these 2 cohorts (TCGA: Spearman *R* = 0.85, *P* < 0.001; ICGC: Spearman *R* = 0.885, *P* < 0.001) (Supplementary Figure 6).

### *MUC16* mutations are associated with immune-active microenvironment

**Figure 3:**
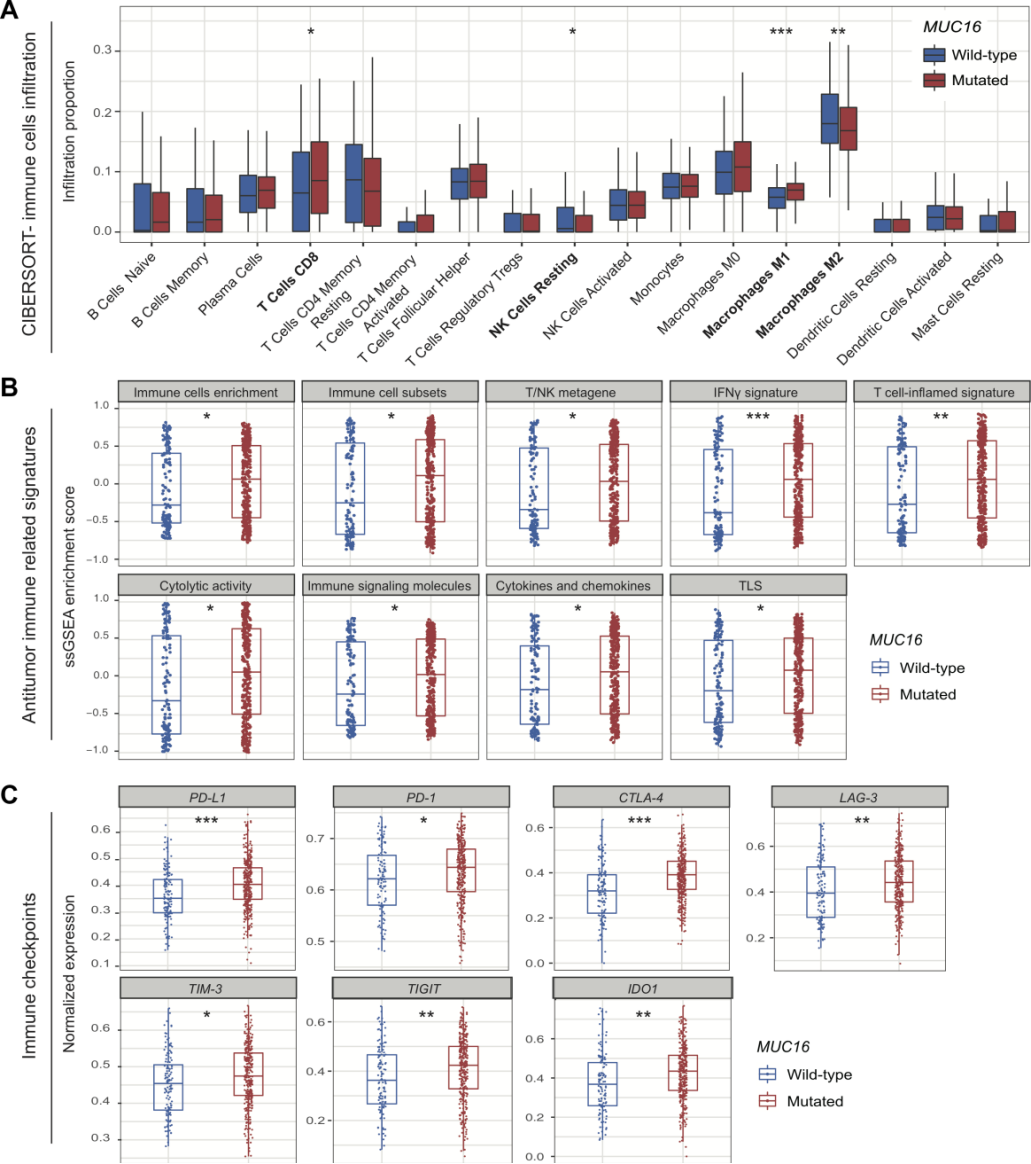
**Association of *MUC16* mutations with tumor microenvironment.** (**A**) Distinct immune cells infiltration in *MUC16* mutated and wild-type subgroups. (**B**) Distribution of immune-related signatures of samples stratified by *MUC16* mutational status. (**C**) Distinct expression of immune checkpoints in *MUC16* mutated and wild-type samples. * *P* < 0.05; ** *P* < 0.01; *** *P* < 0.001.

CIBERSORT method revealed that infiltration of CD8 T cells was significantly higher in patients with *MUC16* mutations (*P* < 0.05), and resting NK cells exhibited the opposite behavior (*P* < 0.05) (Figure 3A). Consistently, we found that patients with *MUC16* mutations had considerably high infiltration of pro-inflammatory M1 macrophages (*P* < 0.001) and low infiltration of immune-suppressive M2 macrophages (*P* < 0.01) (Figure 3A).

Patients with *MUC16* mutations had significant enrichment in total immune cells, immune cell subsets (i.e., T cells, B cells, and NK cells), and T/NK metagene (i.e., T cells and NK cells activity) (all *P* < 0.05; Figure 3B). We also observed high enrichment in IFNγ signal and its related T cell-inflamed gene signature that were previously reported to predict immunotherapy response (both *P* < 0.01; Figure 3B) [16]. Besides, increased cytolytic activity, enrichment in cytokines and chemokines, and enhanced tertiary lymphoid structures (TLS) were all found in patients with *MUC16* mutations (all *P* < 0.05; Figure 3B).

We observed that expression of *PD-L1*, *PD-1,* and *CTLA-4* was significantly upregulated in patients with *MUC16* mutations (Wilcoxon rank sum test, all *P* < 0.05; Figure 3C). Other checkpoints, including *LAG-3*, *TIM-3*, *TIGIT,* and *IDO1* also exhibited consistent results (all *P* < 0.05; Figure 3C).

### Pathways significantly associated with *MUC16* mutations

In gene set enrichment analysis (GSEA) analysis, immune response-related pathways, such as antigen processing and presentation, graft versus host disease, and allograft rejection (normalized enrichment score range: 2.25-2.54; all FDR = 0.001) were among the top enriched pathways of patients with *MUC16* mutations based on KEGG dataset (Supplementary Figure 7A). Pathways for antigen processing and presentation of peptide antigen, response to interferon gamma, and interferon gamma mediated signaling pathway (normalized enrichment score range: 2.46-2.52; all FDR = 0.002) were the top 3 enriched circuits of patients with *MUC16* mutations based on GO dataset (Supplementary Figure 7B).

### *MUC16* mutations are linked with favorable prognosis in 2 cohorts

**Figure 4:**
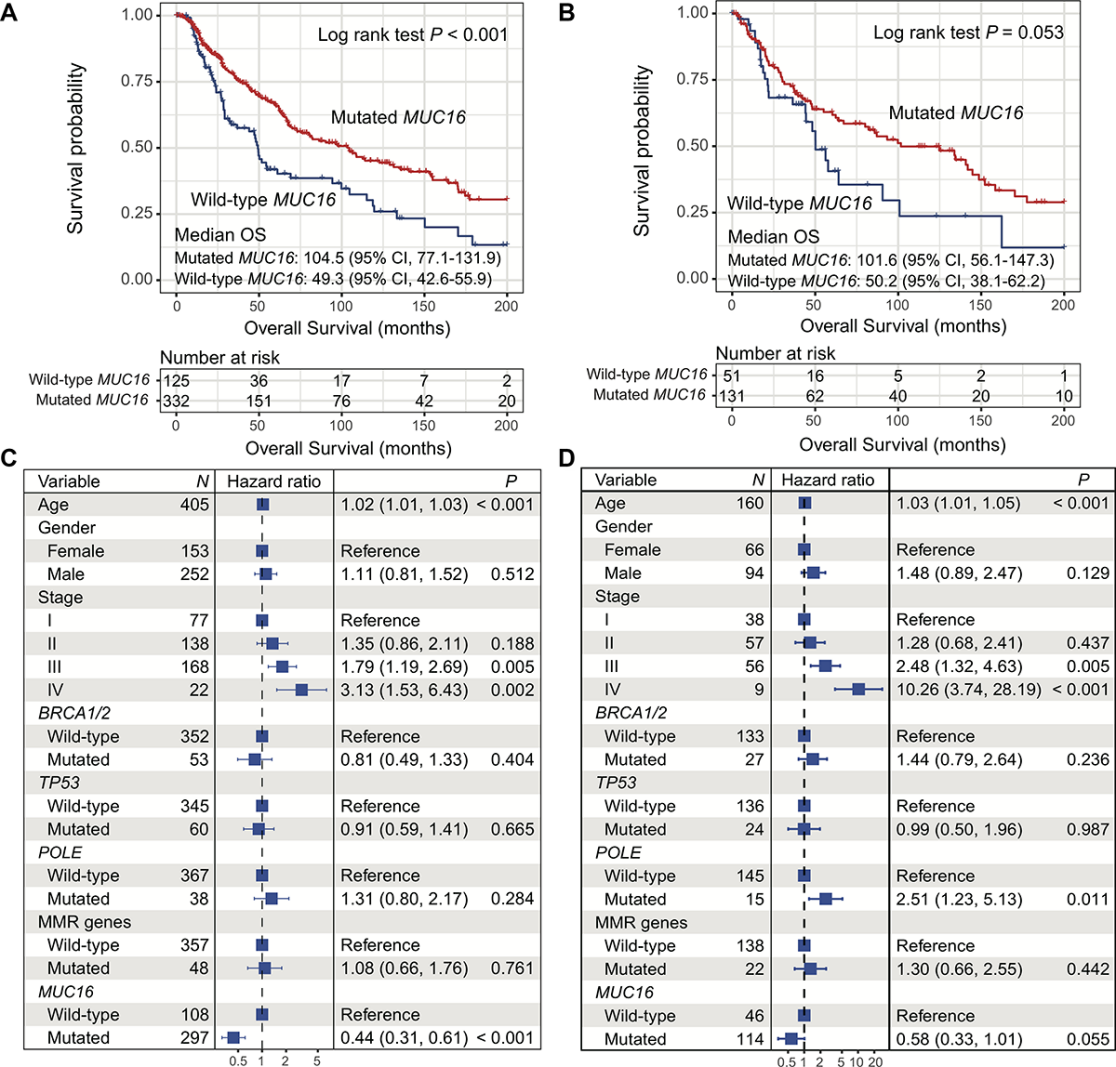
**Correlation of *MUC16* mutations with overall survival in 2 cohorts.** (**A**, **B**) Kaplan-Meier survival analysis based on *MUC16* mutational status (left: TCGA; right: ICGC). (**C**, **D**) Forest plot representation of association of *MUC16* mutations with prognosis (left: TCGA; right: ICGC).

In the TCGA cohort, melanoma patients with *MUC16* mutations had a significantly better overall survival (OS) than those without it (median OS: 104.5 [95% CI, 77.1-131.9] vs. 49.3 [95% CI, 42.6-55.9] months; Log rank test *P* < 0.001; Figure 4A). Multivariate Cox regression model remained statistically significant with confounding factors taken into account (HR: 0.44, 95% CI: 0.31-0.61, *P* < 0.001; Figure 4B).

Consistently, there was a statistically correlation between the presence of *MUC16* mutations and better OS in patients in the ICGC cohort (median OS: 101.6 [95% CI, 56.1-147.3] vs. 50.2 [95% CI, 38.1-62.2] months; Log rank test *P* = 0.053; Figure 4B). The result still remained statistically significant after adjusting for confounding variables (HR: 0.58, 95% CI: 0.33-1.01, *P* = 0.055; Figure 4D).

### *MUC16* mutations were associated with better ICI response and survival in male patients

Consistent with our results for the patients in the TCGA cohort, we found that the presence of *MUC16* mutations were most significantly associated with TML in the ICI-treated cohort (*P* = 5.55E-17; Supplementary Figure 8A). Patients with *MUC16* mutations had significantly higher TML and neoantigen load than those without *MUC16* mutations (median TML: 4.46 vs. 1.53; median neoantigen load: 5.41 vs. 2.36; Wilcoxon rank sum test, both *P* < 0.001) (Supplementary Figure 8B, 8C).

**Figure 5:**
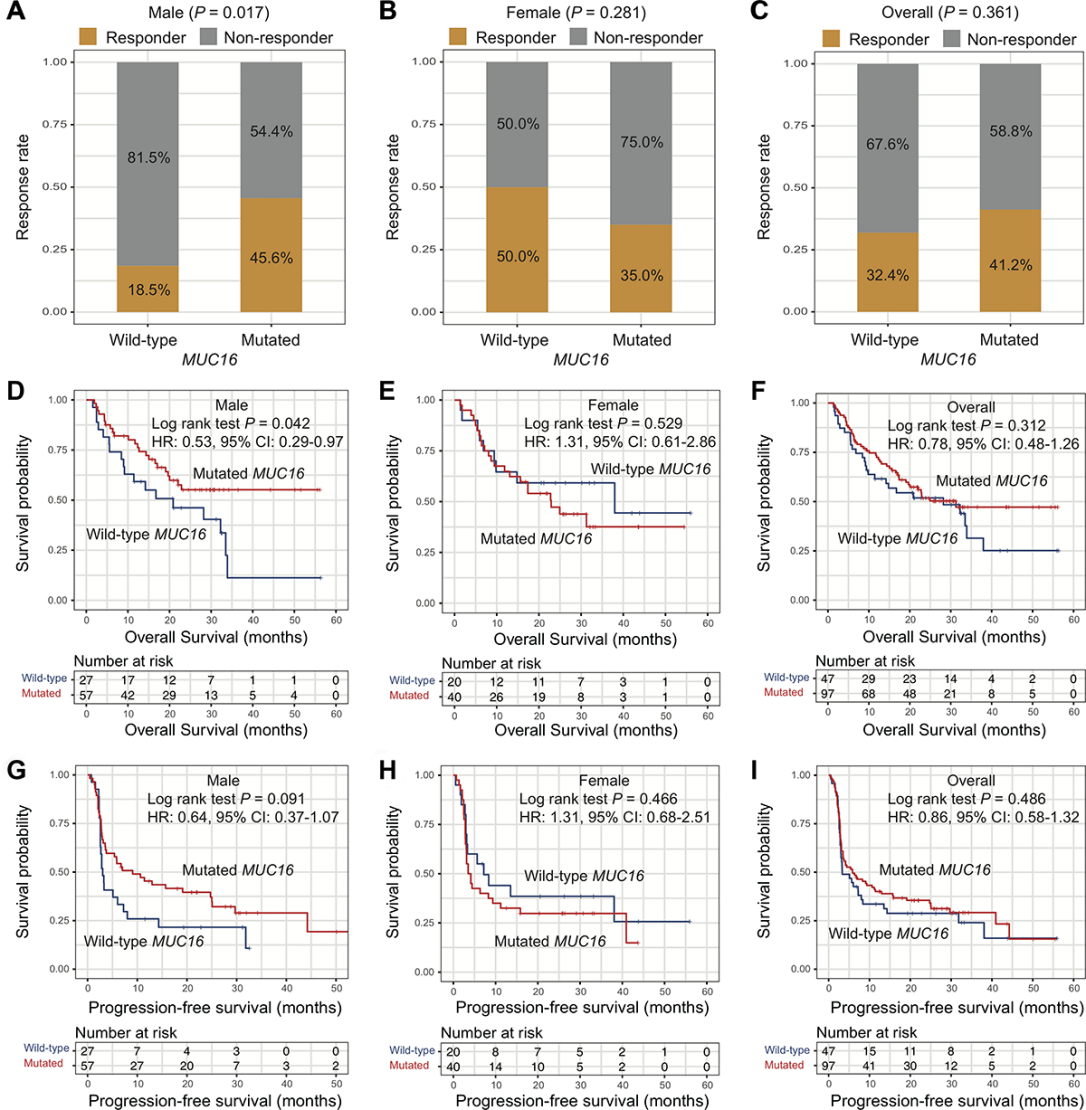
**Association of *MUC16* mutations with ICI therapy efficacy.** (**A**–**C**) Association of *MUC16* mutations with response rate to ICI therapy in male, female, and overall patients. (**D**–**F**) Overall survival plot of ICI treated patients stratified by *MUC16* mutational status in male, female, and overall patients. (**G**–**I**) Progression-free survival plot of ICI treated patients stratified by *MUC16* mutational status in male, female, and overall patients.

In the ICI-treated cohort, patients with *MUC16* mutations had a higher response rate to the therapy than those without *MUC16* mutations in male patients (response rate: 45.6% vs. 18.5%; Fisher exact test, *P* = 0.017; Figure 5A). However, this association was not observed in female patients (response rate: 35.0% vs. 50.0%; Fisher exact test, *P* = 0.281; Figure 5B) and overall patients (response rate: 41.2% vs. 32.4%; Fisher exact test, *P* = 0.361; Figure 5C). Consistent with above findings, we observed that responders had a significantly higher *MUC16* mutation rate than non-responders in male patients (mutation rate: 83.9% vs. 58.5%; Fisher exact test, *P* = 0.017; Supplementary Figure 9A), but not in female patients (mutation rate: 58.3% vs. 72.2%; Fisher exact test, *P* = 0.281; Supplementary Figure 9B) and overall patients (mutation rate: 72.8% vs. 64.0%; Fisher exact test, *P* = 0.361; Supplementary Figure 9C).

Besides, we found that male patients with *MUC16* mutations had a better OS than those without *MUC16* mutations (median OS: not calculable [the median OS of melanoma male patients with *MUC16* mutations could not be calculated owing to more than half patients in this group were alive] vs. 20.9 [95% CI, 9.17-NA (not available)] months; Log rank test *P* = 0.042; Figure 5D). Male patients with *MUC16* mutations exhibited a better trend of progression-free survival (PFS) than those without *MUC16* mutations although this difference did not reach statistical significance (median PFS: 9.07 [95% CI, 3.60-25.1] vs. 3.03 [95% CI, 2.57-8.00] months; Log rank test *P* = 0.091; Figure 5G). In female and overall patients, OS and PFS were not statistically significant when stratified by *MUC16* mutational status (all Log rank test *P* > 0.05) (Figure 5E, 5F, 5H, 5I).

## DISCUSSION

Recently, multiple studies from immunotherapy clinical trials have shown promising findings that harbored a potential to predict or control tumor progression in melanoma [17, 18]. However, inconsistent conclusions of these results highlight the urgent need to determine a more suitable sub-population or biomarkers for immunotherapy in melanoma, which becomes a momentous challenge in the area of immuno-oncology.

Previous clinical trials have revealed that patients with high TML could benefit more from ICI agents in melanoma and non-small cell lung cancer (NSCLC) [19, 20]. However, accurately assessment of TML faces several limitations in clinical practice. The sampling approaches, distinct sequencing platforms, threshold definition, and costs of whole exome sequencing blockade the broadly implementation of TML evaluation [21]. Recent studies have reported that rather than measuring total mutation counts in exome, mutation status of a single specific gene could serve as a surrogate for evaluating TML or ICI therapy efficacy [15, 21–23]. From the TCGA melanoma cohort, we found that patients with *MUC16* mutation had the most significant association with high TML. Noticeably, besides melanoma, our study showed other cancers benefited from immunotherapy (e.g., cancers of lung, colorectal, kidney, bladder, and head and neck) also exhibited the consistent association of *MUC16* mutation with high TML. All these findings suggested patients with *MUC16* mutations may be more responsive to immunotherapies in melanoma and other relevant tumors.

High expression of immune checkpoints such as PD-L1 was approved by FDA as an essential criterion for pembrolizumab treatment in NSCLC [24]. In our study, we found that the other checkpoints (e.g., *PD-1*, *CTLA-4*, *TIM-3* and *LAG-3*) except for *PD-L1*, were all significantly upregulated in patients with *MUC16* mutations. Several studies have reported the immune homeostatic effect of tumor infiltration CD8 T cells and macrophages in tumor-immune microenvironment [25–27]. In this study, we found that patients with *MUC16* mutations had significantly high enrichment in CD8 T cells and M1 macrophages. Conversely, immune suppressive M2 macrophages were enriched in *MUC16* wild-type group. Patients with *MUC16* mutations also harbored a considerably high enrichment of IFNγ and T cell-inflamed signal, which could accurately predict immunotherapeutic efficacy [16, 28]. These findings indicated that *MUC16* mutations were related to immune-activated microenvironment and potentially high response rate to immunotherapy.

Results revealed that the presence of *MUC16* mutations was significantly associated with high ICI response rate and overall survival in male patients from an ICI treated melanoma cohort. This suggests that sex difference may be a potential variable in determining immunotherapeutic efficacy for patients with mutated *MUC16*. Consistent with our findings, a recent meta-analysis reported that male patients gained higher benefit from ICI therapy than female patients (HR: male 0.72 [95% CI, 0.65-0.79] vs. female 0.86 [95% CI, 0.79-0.93]; *P* = 0.002) [29]. In our study, we found that male patients harbored significantly higher TMB than female patients (Figure 2C), which was also reported in a previous study [30]. This finding suggested that male patients may have higher neoantigen and immunogenicity, which speculatively justifies association of *MUC16* mutation with higher response rate and better ICB therapy outcome in male patients as compared with female patients.

Several limitations remained in our study. Firstly, melanoma samples with gene expression profile were only acquired from one cohort. Secondly, the mechanisms for correlation between *MUC16* mutations and higher mutation load are elusive, which requires further investigation.

Our study discovered that patients with *MUC16* mutations had significantly high TML, immune-activated tumor microenvironment and favorable survival outcome. Importantly, the presence of *MUC16* mutations was significantly associated with better immunotherapeutic efficacy in male patients. Therefore, *MUC16* mutations may serve as a surrogate for predicting efficacy of immune checkpoints based therapies, and future clinical trials are needed to validate our findings.

## MATERIALS AND METHODS

### Genomic and clinical information of melanoma patients

Somatic mutation data of 467 melanoma samples in the Cancer Genome Atlas (TCGA) cohort were acquired from Genome Data Commons (https://portal.gdc.cancer.gov). The validation dataset contained 183 samples and was obtained from International Cancer Genome Consortium (ICGC) (https://dcc.icgc.org). Gene expression profiles of 465 patients were obtained from TCGA cohort.

The ICI therapy cohort was obtained from the study by Liu et al. [31], which is the largest publicly available melanoma ICI-treated patient cohort. This cohort contained 144 patients treated with either anti-PD-1 or anti-CTLA-4 agents. In this study, patients with complete or partial response were considered responders; other statuses (i.e., progressive disease, stable disease, and mixed response) were considered non-responders.

### *MUC16* mutation versus TML

Genomic instability or high mutation load is largely correlated with mutations in DNA damage repair (DDR) and mismatch repair (MMR) related genes [32]. In addition to univariate analysis of association of *MUC16* mutations with TML, we performed multivariate logistic regression with mutations in DDR genes (e.g., *BRCA1/2*, *TP53,* and *POLE*) and MMR genes (i.e., *MLH1*, *MSH2*, *MSH6,* and *PMS2*), and clinical factors as confounding variables to eliminate the false positive possibility. TML was defined as log2 transformation of mutation counts per megabase. We applied a univariate clustering approach (i.e., *Ckmeans.1d.dp* algorithm) available from *R* package *Ckmeans.1d.dp* (version 4.2.2) [33] to determine the optimal cutoff value of high versus low TML followed by recently broadly used value (i.e., 17 mutation counts per megabase, log2 transformed level [4.09]).

### Microenvironment-based cellular and immune-related signatures

Estimation of tumor infiltration immune cells was performed using CIBERSORT algorithm with the LM22 signature [34]. In this study, we analyzed only 17 immune cell types owing to the less enrichment of the other 5 cell types (i.e., naive CD4 T cell, gamma delta T cells, activated mast cells, eosinophils, and neutrophils).

Previously reported representative immune-related signatures that indicated distinct immune cells and statuses were curated as follows: 1) immune cells enrichment, which indicates total immune cells infiltration in tumor microenvironment [35]; 2) immune cell subsets, enrichment of T cells, B cells and NK cells [2]; 3) T/NK metagene, which reflects the activity of T cells and NK cells [36]; 4) IFNγ signature, a signal located in the central site of antitumor immune response and that correlates with immunotherapy response [37]; 5) T cell-inflamed signature, which is comprised of 18 inflammatory genes associated with immune response [28]; 6) immune cytolytic activity [38]; 7) immune signaling molecules [2]; 8) cytokines and chemokines [2]; and 9) TLS, the ectopic lymphoid formations associated with inflammation response [39].

Immune checkpoints in melanoma primarily include PD-L1, PD-1, and CTLA-4 [40, 41]. Additional checkpoints, for example, LAG-3, TIM-3, TIGIT, and IDO1, are being tested in clinical trials and play crucial roles in immunotherapy [42–45]. We therefore compared differential expression of these genes according to *MUC16* mutational status.

### Gene set enrichment analysis

We applied single sample gene set enrichment analysis (ssGSEA) approach embedded in *R* package *GSVA* (version 1.32.0) to evaluate overall enrichment of specific immune signatures of each sample [46]. GSEA was implemented by *fgsea* package (version 1.10.0). Signaling pathways in Kyoto Encyclopedia of Genes and Genomes (KEGG) and Gene Ontology (GO) were used as the background database.

### Statistical analyses

*R* software (version 3.6.1) was used in this study to perform relevant statistical analyses. Mutation patterns were presented via *GenVisR* package (version 1.16.0) [47]. We drew and compared survival curves using Kaplan-Meier approach and Log rank test respectively with *R* *survival* (version 2.44-1.1) and *survminer* (version 0.4.5) packages. Multivariate logistic and Cox regression models were built using *forestmodel* package (version 0.5.0). Associations of *MUC16* mutations with continuous and categorical variables were estimated with Wilcoxon rank sum test and Chi-square test, separately.

## Supplementary Material

Supplementary Figures

Supplementary Table 1
