# Supplementary material for: High mutation load, immune-activated microenvironment, favorable outcome, and better immunotherapeutic efficacy in melanoma patients harboring MUC16/CA125 mutations
Source: Aging (Albany NY). 2020 Jun 3;12(11):10827–43. doi: 10.18632/aging.103296 (PMC7346065; doi:10.18632/aging.103296)
Supplement: Supplementary Figures [file aging-12-103296-s002..pdf]

SUPPLEMENTARY FIGURES

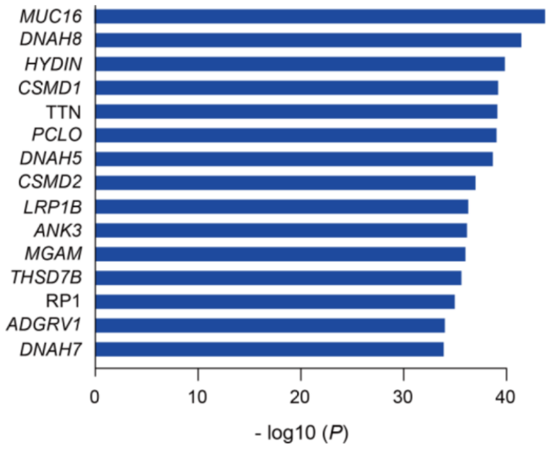

Supplementary Figure 1. The top 15 significant association of single gene mutation with mutation load in TCGA cohort.

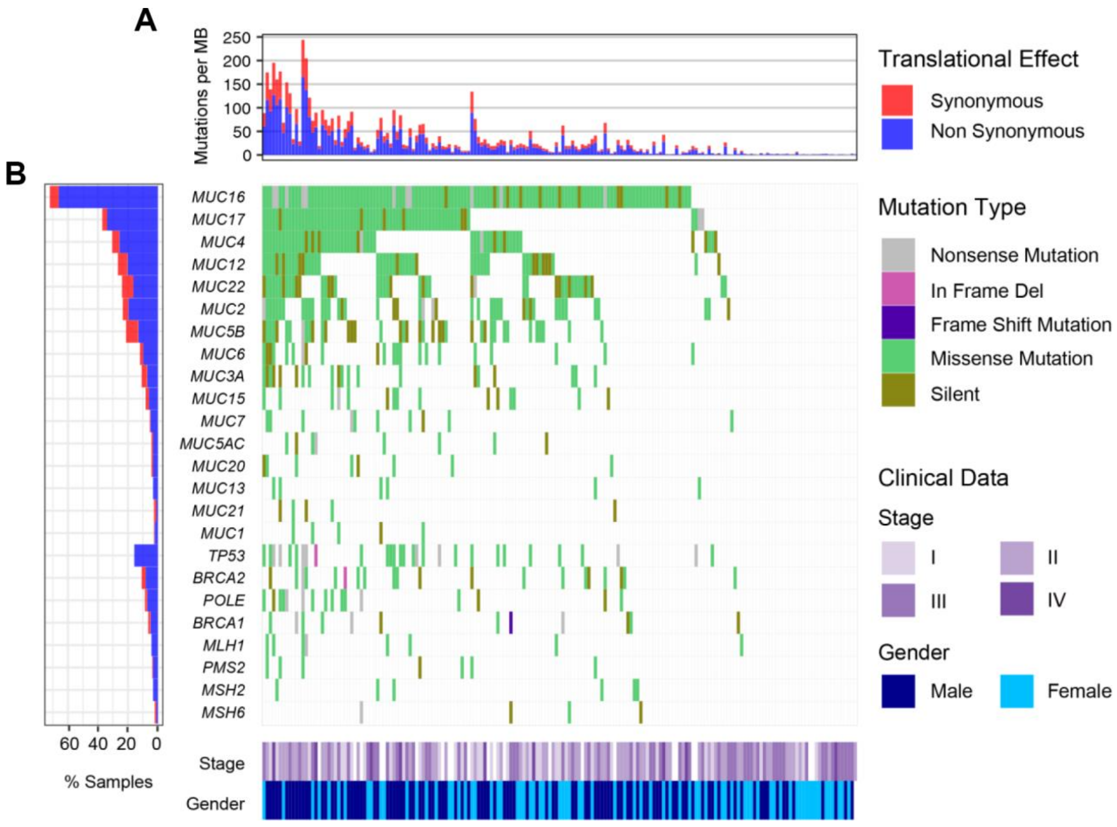

Supplementary Figure 2. Mutational patterns of *MUC16* and mucin family members in relation to DNA repair-related genes in ICGC cohort. (**A**) Numbers of mutations per megabase in each sample. (**B**) Representation for mutation patterns of mucin and DNA repair genes.

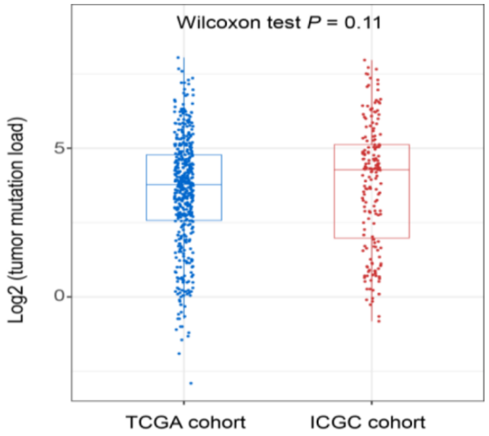

Supplementary Figure 3. Comparison of TML between TCGA and ICGC cohorts.

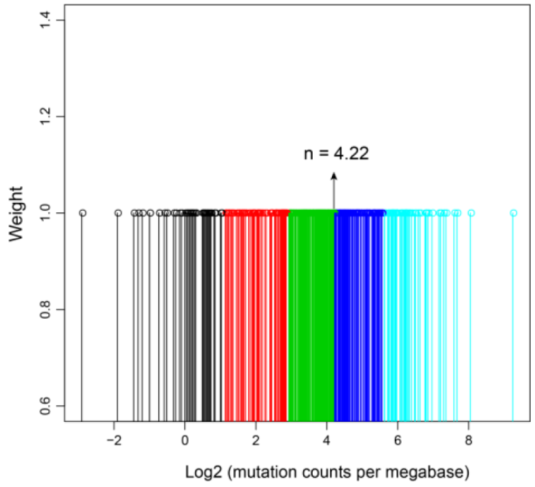

Supplementary Figure 4. Univariate k-means clustering of TML.

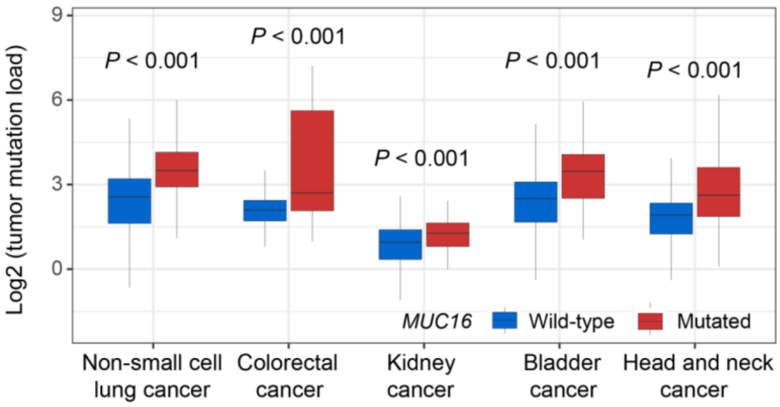

Supplementary Figure 5. Differences of TML in patients with and without *MUC16* mutations in other 5 immunotherapy susceptible cancers in TCGA.

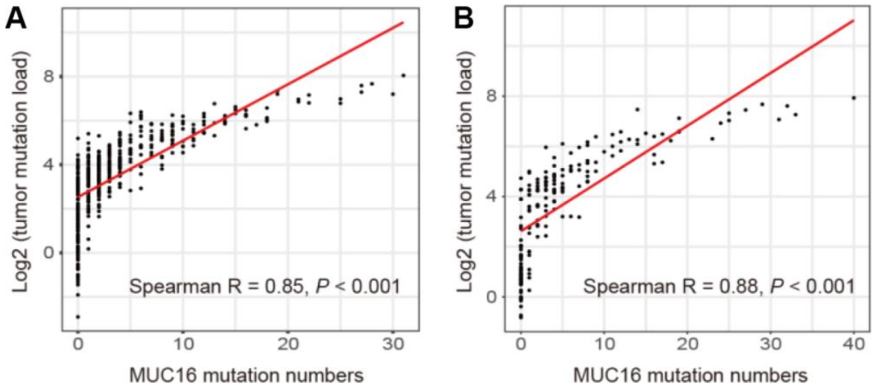

Supplementary Figure 6. Association of *MUC16* mutation numbers with TML in 2 melanoma cohorts (left: TCGA; right: ICGC).

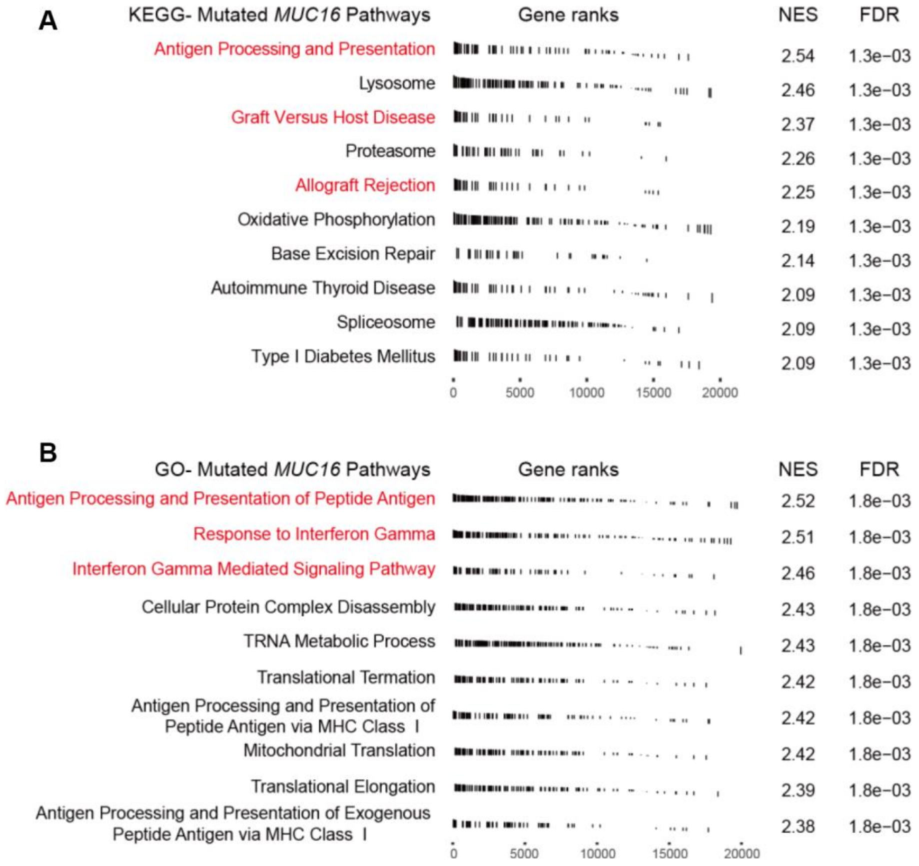

Supplementary Figure 7. Top enriched pathways of patients with *MUC16* mutations in (**A**) KEGG and (**B**) GO.

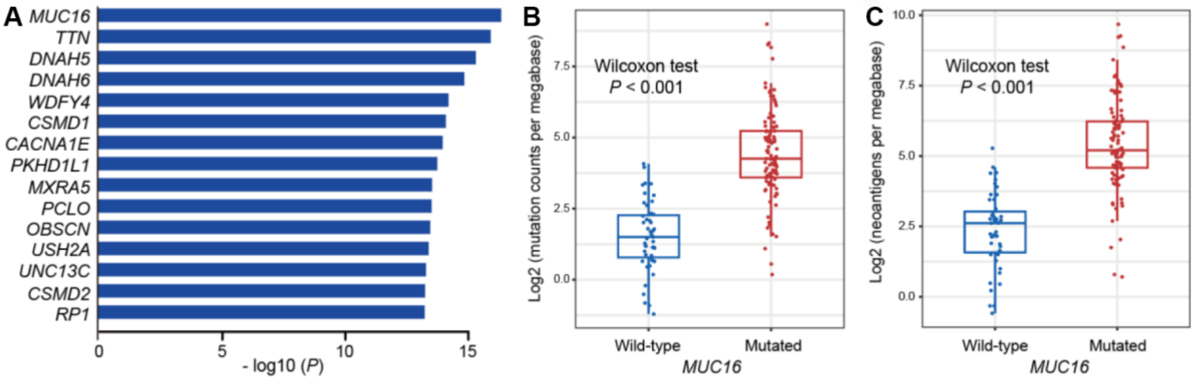

Supplementary Figure 8. Association of *MUC16* mutation with TML and neoantigen load in the ICI treated cohort. (**A**) Top 15 significant association of single gene mutation with TML. (**B**) Association of *MUC16* mutation with TML. (**C**) Association of *MUC16* mutation with neoantigen load.

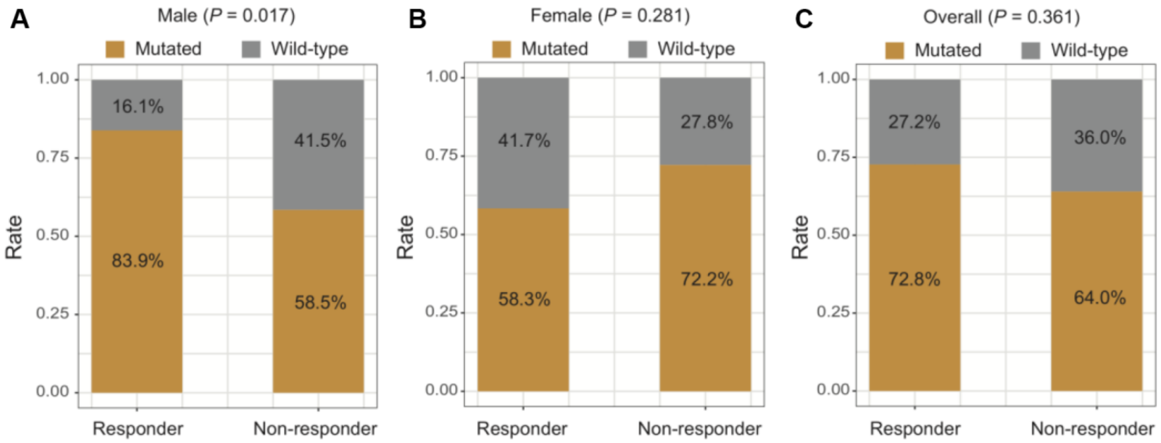

Supplementary Figure 9. Differences of *MUC16* mutation rate between responders and non-responders in (**A**) male patients, (**B**) female patients and (**C**) overall patients.
