# Supplementary material for: High mutation load, immune-activated microenvironment, favorable outcome, and better immunotherapeutic efficacy in melanoma patients harboring MUC16/CA125 mutations
Source: Aging (Albany NY). 2020 Jun 3;12(11):10827–43. doi: 10.18632/aging.103296 (PMC7346065; doi:10.18632/aging.103296)
Supplement: Supplementary Table 1 [file aging-12-103296-s001..pdf]

SUPPLEMENTARY TABLE

Supplementary Table 1. Cooccurrent mutation of *MUC16* and DNA repair genes.

	TCGA cohort			ICGC cohort		
	MUC16		***P*** **value**	*MUC16*		***P*** **value**
	**Mutated**	**Wild-type**		**Mutated**	**Wild-type**	
TP53						
Mutated	57 (16.7%)	7 (5.6%)	0.001	27 (20.5%)	2 (3.9%)	0.006
Wild-type	284 (83.3%)	119 (94.4%)		105 (70.5%)	49 (96.1%)	
POLE						
Mutated	44 (12.9%)	1 (0.8%)	< 0.001	16 (12.1%)	0 (0.0%)	0.007
Wild-type	297 (87.1%)	125 (99.2%)		116 (87.9%)	51 (100.0%)	
BRCA1/2						
Mutated	56 (16.4%)	5 (4.0%)	< 0.001	26 (19.7%)	2 (3.9%)	0.006
Wild-type	285 (83.6%)	121 (96.0%)		106 (80.3%)	49 (96.1%)	
MMR genes						
Mutated	49 (14.4%)	2 (1.6%)	< 0.001	22 (16.7%)	0 (0.0%)	0.001
Wild-type	292 (85.6%)	124 (98.4%)		110 (83.3%)	51 (100.0%)	
